# Varying Atmospheric CO_2_ Mediates the Cold-Induced CBF-Dependent Signaling Pathway and Freezing Tolerance in Arabidopsis

**DOI:** 10.3390/ijms21207616

**Published:** 2020-10-15

**Authors:** Jinyoung Y. Barnaby, Joonyup Kim, Mura Jyostna Devi, David H. Fleisher, Mark L. Tucker, Vangimalla R. Reddy, Richard C. Sicher

**Affiliations:** 1 Adaptive Cropping Systems Laboratory, Agricultural Research Service, USDA, Building 001, 10300 Baltimore Ave., Beltsville, MD 20705, USA; jyostna.mura@usda.gov (M.J.D.); david.fleisher@usda.gov (D.H.F.); Vangimalla.reddy@usda.gov (V.R.R.); rsicher1981@gmail.com (R.C.S.); 2 Dale Bumpers National Rice Research Center, Agricultural Research Service, USDA, Building 001, 10300 Baltimore Ave., Beltsville, MD 20705, USA; 3 Soybean Genomics and Improvement Laboratory, Agricultural Research Service, USDA, Building 006, 10300 Baltimore Ave., Beltsville, MD 20705, USA; joonyup.kim@gmail.com (J.K.); mark.tucker@usda.gov (M.L.T.)

**Keywords:** abiotic stress, carbon dioxide, atmospheric CO_2_, C-repeat binding factor, cold-responsive gene, stomatal regulation, freezing tolerance

## Abstract

Changes in the stomatal aperture in response to CO_2_ levels allow plants to manage water usage, optimize CO_2_ uptake and adjust to environmental stimuli. The current study reports that sub-ambient CO_2_ up-regulated the low temperature induction of the C-repeat Binding Factor (CBF)-dependent cold signaling pathway in Arabidopsis (*Arabidopsis thaliana*) and the opposite occurred in response to supra-ambient CO_2_. Accordingly, cold induction of various downstream cold-responsive genes was modified by CO_2_ treatments and expression changes were either partially or fully CBF-dependent. Changes in electrolyte leakage during freezing tests were correlated with CO_2′_s effects on CBF expression. Cold treatments were also performed on Arabidopsis mutants with altered stomatal responses to CO_2_, i.e., high leaf temperature 1-2 (*ht1-2*, CO_2_ hypersensitive) and β-carbonic anhydrase 1 and 4 (*ca1ca4*, CO_2_ insensitive). The cold-induced expression of CBF and downstream CBF target genes plus freezing tolerance of *ht1-2* was consistently less than that for Col-0, suggesting that HT1 is a positive modulator of cold signaling. The *ca1ca4* mutant had diminished CBF expression during cold treatment but the downstream expression of cold-responsive genes was either similar to or greater than that of Col-0. This finding suggested that βCA1/4 modulates the expression of certain cold-responsive genes in a CBF-independent manner. Stomatal conductance measurements demonstrated that low temperatures overrode low CO_2_-induced stomatal opening and this process was delayed in the cold tolerant mutant, *ca1ca4*, compared to the cold sensitive mutant, *ht1-2*. The similar stomatal responses were evident from freezing tolerant line, Ox-CBF, overexpression of *CBF3,* compared to wild-type ecotype Ws-2. Together, these results indicate that CO_2_ signaling in stomata and CBF-mediated cold signaling work coordinately in Arabidopsis to manage abiotic stress.

## 1. Introduction

Cold stress (freezing or chilling temperatures) is one of the significant environmental stresses that can severely impact crop yields and limit plant species’ geographical distribution. Consequently, many terrestrial plants have developed strategies to survive prolonged exposures to low and freezing temperatures [1]. Cold acclimation in higher plants involves various physiological and molecular changes that enhance freezing tolerance regulated by both CBF (C-repeat-binding factors)-dependent and -independent pathways [2]. Transcripts of *CBF* family genes are induced in response to a cold shock [3,4,5], and CBF proteins subsequently bind to the CRT/DRE (C-repeat/dehydration response element) *cis*-acting element in the promoter regions of *COLD-RESPONSIVE* (*COR*), *EARLY DEHYDRATION INDUCIBLE* (*ERD*), and other cold related genes, such as *RESPONSIVE TO DEHYDRATION* (*RD*) and *COLD-INDUCIBLE* (*KIN*) [6,7,8]. In Arabidopsis and other plant species, cold stress induces the expression of CBF transcription factors, which subsequently activate downstream molecular cascades of *COR* genes [9,10,11]. Transcriptome profiling in Arabidopsis showed induction of about 400 *COR* genes after 24 h treatment at 4 °C regulated by *CBF1, CBF2*, and *CBF3,* playing a significant role in cold acclimation [11]. In a recent study, more than 3000 *COR* genes displayed a significant change in their expression levels in the mutants of *CBF1* to *CBF3* compared to the wild-type plants after being treated at 4 °C for 12 h [12]. However, only ~12% of the cold-responsive genes are controlled by CBFs [13]. Therefore, some CBF-independent cold signaling pathways such as ABA-dependent cold signaling, RD22, RD29A, chalcone synthase, and other phytohormones function in cold signaling to produce *COR* genes [14,15,16]. Proteins synthesized from *COR* genes act as cryoprotectants and enhance low temperature tolerance, and the ability of plants to survive episodes of frost [3,4,17,18,19,20].

Stomatal movement in plants is controlled by diverse factors that include CO_2_, blue light, photoperiod, fusicoccin, abscisic acid, calcium ions, and temperature [21]. In general, the stomatal aperture responds inversely to CO_2_ concentrations in the air by elaborate CO_2_ sensing and signaling mechanisms [22]. The relationship between stomatal aperture and cold tolerance/winter hardiness was recognized in the late 1970s [23,24]. It was shown that cold-tolerant plants attained improved leaf water potential by closing stomata and limiting water loss during prolonged exposure to low temperatures [23]. Cold-sensitive species tended to have more open stomata when exposed to freezing temperatures and were more susceptible to desiccation, exhibiting low water potential [24,25]. In Arabidopsis, the gene *Inducer of CBF Expression 1* (*ICE1*) is a transcription factor binding to CBF promoters and other regulatory genes, and initiates the CBF-dependent cold signaling pathway known to be critical for the cold response [25,26,27]. In a separate study of genes that regulate stomatal development in Arabidopsis, the stomatal-associated gene, called *SCREAM* (*SCRM*), was demonstrated to be the same gene as *ICE1* [28]. Moreover, the protein of the *SPEECHLESS* (*SPCH*) transcription factor, which is a critical integrator of developmental and environmental signals affecting stomatal initiation and proliferation, binds to the promoters for *ICE1*/*SCRM* and other related genes [29,30,31,32]. The *ICEI/SCRM* double knockout mutant developed an epidermis devoid of stomata and the gain-of-function mutant *SCRM-D* (dominant mutant) elevated expression of the stomatal gene *EPF* (epidermal patterning factor) and produced an epidermis solely composed of stomata [28,32,33]. These independent observations linking cold signaling and stomatal development supported a role for regulation of stomatal development in cold tolerance. Engineer et al. (2014) [34] demonstrated a direct CO_2_ effect on stomatal development. These authors showed that *EPF* is necessary for CO_2_ controlled stomatal development, and the expression of *EPF2* is increased by high CO_2_ in a βCA1/4-dependent manner. Together, these studies suggested a connection exists between stomatal aperture and cold responses in plants.

Two Arabidopsis mutants that disrupt the CO_2_-induced stomatal movement have been identified previously [35,36]. The *ht1-2* (high leaf temperature) mutant has closed stomata when exposed to sub-ambient CO_2_ concentrations (hypersensitive stomatal response). However, the *ht1-2* mutant still responded to other stomatal signals, such as blue light, fusicoccin and abscisic acid, suggesting that HT1 is an important regulator of CO_2_ signaling in the control of stomatal aperture [36]. Conversely, the *ca1ca4* double mutant [35] displayed increased stomatal density and the stomata did not close in response to supra-ambient CO_2_ (insensitive stomatal CO_2_ response). Thus, the Arabidopsis mutants described above mimicked stomatal responses to high and low CO_2_ conditions, respectively, and we hypothesized that these mutants could be used as genetic tools to understand molecular responses to cold treatments.

Here, we report that varying CO_2_ levels in the air altered the low-temperature expression of three *CBF* genes and selected downstream cold-responsive genes. Our findings were further supported using mutants with impaired stomatal responses to CO_2_. In addition, changes in the expression of cold-responsive genes by CO_2_ were positively correlated with freezing tolerance as measured by electrolyte leakage.

## 2. Results

### 2.1. Varying Atmospheric CO_2_ Modulates the CBF-Mediated Cold Signaling Pathway

**Figure 1:**
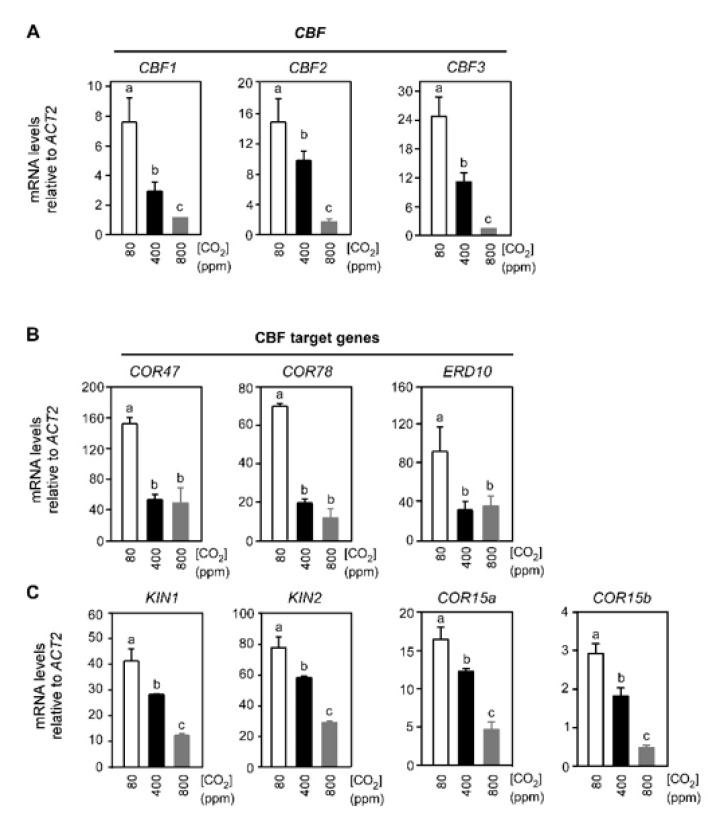
The low temperature expression of *CBF* and downstream CBF-dependent genes in Arabidopsis is modified by air levels of CO_2_. Individual 3.5 week-old Arabidopsis plants (Col-0) were exposed to 4 °C temperature for 2 h with sub-ambient, ambient, or supra-ambient CO_2_, (80, 400 and 800 μmol mol^−1^, respectively). Transcript abundance of three *CBF* genes (**A**), and seven downstream CBF target genes (**B** and **C**) were determined by q-PCR. The expression of measured transcripts was normalized to *ACT2*. Relative values are means (×1000) of three biological replicates and two technical replicates (error bars indicate SEM). Bars labeled with different lowercase letters differed at *p* ≤ 0.05 and differences were determined using the one-way analysis of variance procedure (ANOVA) and Tukey HSD.

Initially, we examined the effects of supra-ambient, ambient, and sub-ambient CO_2_ (800, 400, and 80 μmol mol^−1^, respectively) on changes in expression of *CBF1, CBF2,* and *CBF3* in wild-type Arabidopsis (Col-0) in response to 2 h of exposure to 4 °C. The expression of all three *CBF* genes was most significant when plants were exposed to sub-ambient CO_2_. The expression levels decreased proportionally (*p* ≤ 0.01) in response to supra-ambient CO_2_ (Figure 1A). Later, we observed the effect of varying atmospheric CO_2_ on downstream CBF-dependent target genes in the cold signaling pathway, i.e., *COR47* (*RD17*), *COR78* (*RD29A*/*LTI78*), *ERD10* (*LTI45*/*LTI29*), *KIN1, KIN2* (*COR6.6*), *COR15a*, and *COR15b* (Figure 1B,C). The low temperature-dependent expression for *COR47*, *COR78*, and *ERD10* was enhanced (*p* ≤ 0.01) at sub-ambient compared to ambient CO_2_, but the expression of these same three genes did not differ (*p* > 0.5) between the ambient and supra-ambient CO_2_ treatments (partially CO_2_ dependent, Figure 1B). The low temperature-induced expression of *KIN1*, *KIN2*, *COR15a*, and *COR15b* was greatest (*p* ≤ 0.01) at sub-ambient and least at supra-ambient CO_2_ (fully CO_2_ dependent, Figure 1C).

### 2.2. Varying Atmospheric CO_2_ Modifies the Freezing Tolerance of Arabidopsis

**Figure 2:**
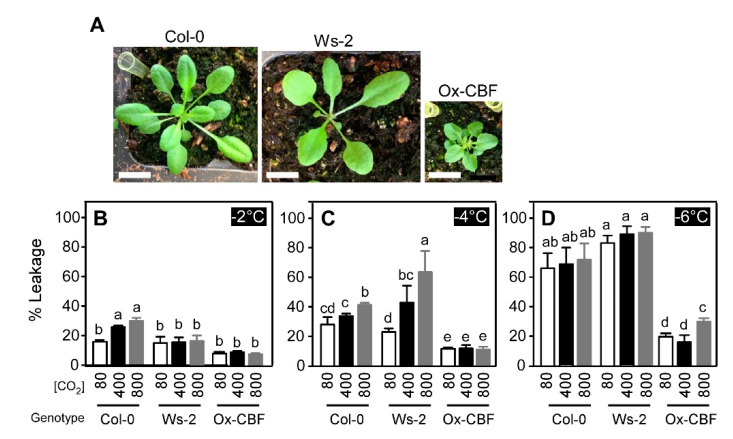
Electrolyte leakage due to freezing temperatures in Arabidopsis was altered in response to CO_2_ treatments. Individual 3.5 week-old wild type Arabidopsis plants ecotypes (ecotypes Col-0 and Ws-2) and the CBF3 over-expression (Ox-CBF) plant (**A**) were initially incubated at 4 °C for 3 h at sub-ambient, ambient or supra-ambient CO_2_ (80, 400 and 800 μmol mol^−1^, respectively). The chamber temperature was then lowered at 1 °C h^−1^ and plants were sampled for electrolyte leakage at −2 (**B**), −4 (**C**), and −6 °C (**D**). Electrolyte leakage into deionized water was measured as changes in conductivity and the percentages shown are mean values from three biological replicates and two technical replicates (error bars indicate SEM). Bars labeled with different lowercase letters differed at *p* ≤ 0.05 and differences were determined using Two-way ANOVA and Tukey HSD. White scale bars in (**A**): 0.5 cm.

The electrolyte leakage was measured using two ecotypes of Arabidopsis, Col-0 and Ws-2, to determine the effects of changing CO_2_ concentration on CBF-mediated cold signaling altered freezing tolerance (Figure 2A). In addition, a plant that over-expresses *CBF3* (Ox-CBF) in the Ws-2 background [37], which constitutively activates downstream cold signaling cascades (Figure 2A), was included for the comparison. At −2 °C, 3.5-week-old Col-0 plants displayed 17% electrolyte leakage at sub-ambient CO_2_ (*p* ≤ 0.01) and this increased to 26 and 30% leakage at ambient and supra-ambient CO_2_, respectively (Figure 2B). The response of electrolyte leakage by Col-0 plants to changing CO_2_ was similar at −2 and −4 °C, but the total amount of electrolyte leakage was greater at the lower temperature (Figure 2B,C). Interestingly, no significant differences in electrolyte leakage in response to CO_2_ at −2 °C were observed using the Ws-2 ecotype (*p* > 0.5). However, at −4 °C, the electrolyte leakage was 2.7-fold greater (*p* ≤ 0.01) at supra-ambient than at sub-ambient CO_2_ (Figure 2B,C). When the temperature was lowered to −6 °C, the CO_2_ effects on electrolyte leakage did not differ between Col-0 and Ws-2 (*p* > 0.05), which indicated that both ecotypes were approaching maximal electrolyte leakage (Figure 2D). Interestingly, the freezing-tolerant Ox-CBF plant displayed somewhat unexpected results. Electrolyte leakage from Ox-CBF remained unaffected by the change in CO_2_ levels (*p* > 0.5) at −2 and −4 °C (Figure 2B,C). However, significant differences (*p* ≤ 0.01) in electrolyte leakage were observed at supra-ambient CO_2_ treatments at −6 °C compared to sub-ambient and ambient CO_2_ treatments. In addition, electrolyte leakage was more than 50% less in the Ox-CBF line compared to Ws-2 (Figure 2D), irrespective of CO_2_ treatment. This indicated a possible role for CBF-mediated signaling in CO_2_-dependent responses of cold-induced electrolyte leakage. The above results suggest that the changes in CO_2_ level in air modified the freezing tolerance of Arabidopsis via CBF-mediated cold signaling (Figure 1 and Figure 2).

### 2.3. Arabidopsis Mutants Defective in Stomatal Responses to CO_2_ Exhibit Altered CBF-Mediated Cold Signaling

**Figure 3:**
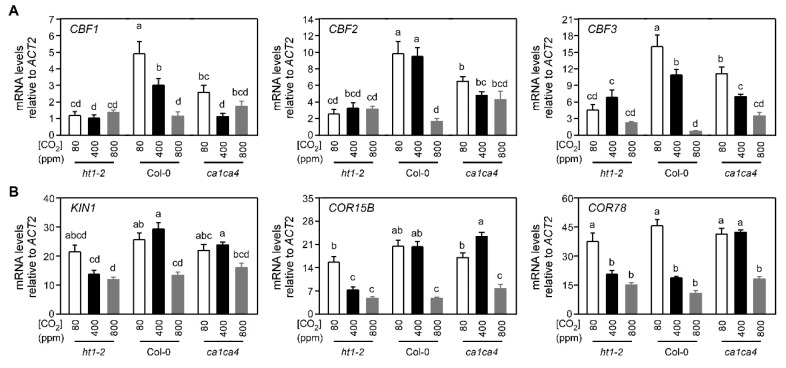
The low temperature expression of CBFs (**A**) and downstream CBF-dependent genes (**B**) under varying CO_2_ levels (800, 400, and 80 μmol mol^−1^) was altered in two Arabidopsis CO_2_ stomatal response mutants along with wildtype control, Col-0. Transcript abundance was determined using Arabidopsis (Col-0) plants and two stomatal response mutants that were hypersensitive or insensitive to CO_2_ (i.e., *ht1-2* and *ca1ca4*, respectively). Individual 3.5-week-old plants grown at 22 °C were exposed to 4 °C temperature for 2 h under ambient CO_2_ conditions. Relative values are means (×1000) of three biological replicates and two technical replicates (error bars indicate SEM). Bars labeled with different lowercase letters differed at *p* ≤ 0.05 and differences were determined using two-way ANOVA and Tukey HSD. This experiment was performed independently from the one in Figure 1; therefore, the data values are not the same, but a similar trend was observed.

Considering the observations above, we further investigated whether stomatal response mutants mimicking supra- and sub-ambient CO_2_ conditions would show similar changes in CO_2_ control through CBF-mediated cold signaling. For these experiments, we used the *ht1-2* mutant, hypersensitive to CO_2_, and the *ca1ca4* double mutant, whose stomata are insensitive to CO_2_ and remain open regardless of CO_2_ levels (Figure 3). All CBFs and CBF target genes tested here were significantly upregulated in Col-0 and in the *ht1-2*, and *ca1ca4* mutants in response to cold treatment (i.e., 0 h vs. 2 h) (Figure S2). These measurements were performed using 3.5-week-old plants exposed to supra-ambient, ambient, and sub-ambient CO_2_ (800, 400, and 80 μmol mol^−1^) and maintained at 22 °C (0 h) or exposed to 4 °C for 2 h. The cold induced expression levels of *CBF* genes significantly decreased in response to increasing CO_2_ levels in Col-0 (Figure 3A). This CO_2_-dependent, cold stress-triggered change in CBF expression was abolished in the *ht1-2* mutant, which closes stomata when exposed to sub-ambient CO_2_ concentrations (Figure 3A). In contrast, cold-induced CBF expression at supra-ambient CO_2_ conditions was greater in the *ca1ca4* mutant, which does not close stomata under supra-ambient CO_2,_ compared to Col-0 (Figure 3A). This result indicates the involvement of molecular components (i.e., βCA1/4 and HT1) in CO_2_ control of stomatal movement. The changes in the atmospheric CO_2_ conditions did not have a significant effect on the basal expression of the CBF genes measured at room temperature (Figure S3).

Next, we explored the link between the regulation of stomatal aperture and the expression of downstream CBF target genes. Interestingly, the two groups of genes, i.e., either partially (Figure 1B) or fully (Figure 1C) CO_2_-dependent, responded differently in the *ht1-2* and *ca1ca4* mutants under ambient CO_2_ condition (Figure S2B,C). The cold-induced expression of *KIN1*, *KIN2*, *COR15a*, and *COR15b* that were fully CO_2_-dependent was decreased in the *ht1-2* mutant relative to Col-0 (Figure S2B,C), while that of *COR47*, *COR78*, and *ERD10,* that were partially CO_2_-dependent (Figure 1B), was greater (*p* ≤ 0.05) in the *ca1ca4* mutant than in Col-0 (Figure S2B). Enhanced expression of the partially CO_2_ dependent CBF target genes and reduced expression of the fully CO_2_-dependent CBF target genes in the *ca1ca4* mutant compared to their expression in the Col-0 wild type was observed (Figure S2B). Furthermore, we studied the low-temperature expression of cold-regulated genes that are independent of CBF regulation, i.e., *ARABIDOPSIS THALIANA HOMEOBOX PROTEIN 2* (*ATHB2*), *RD22*, and *CHALCONE SYNTHASE* (*CHS*). The cold-induced expression of *ATHB2* and *RD22* in the Col-0 plants were not significantly induced by sub-ambient CO_2_ treatment. However, the expression levels of *CHS* were significantly higher in the *ca1ca4* mutant, and sub-ambient CO_2_-treated Col-0 compared to ambient CO_2_-treated Col-0 plants (*p* < 0.05 to *p* < 0.01) (Figure S1).

We further explored CO_2′_s effect on cold-induced expression of CBF target genes, i.e., *KIN1*, *COR15b*, and *COR78*. In general, the cold-induced expression of these CBF target genes decreased in response to rising CO_2_ levels in Col-0 and two stomatal response mutants, *ht1-2* and *ca1ca4* (Figure 3B). However, this CO_2_-dependent, cold stress-triggered CBF target gene expression was reduced in the *ht1-2* mutant compared to Col-0. In contrast, the cold-induced *KIN1* expression at supra-ambient CO_2_ conditions was slightly greater in the *ca1ca4* mutant compared to Col-0, except *COR78* (Figure 3B).

### 2.4. Defects in Stomatal Responses to CO_2_ Alter Freezing Tolerance in Arabidopsis

**Figure 4:**
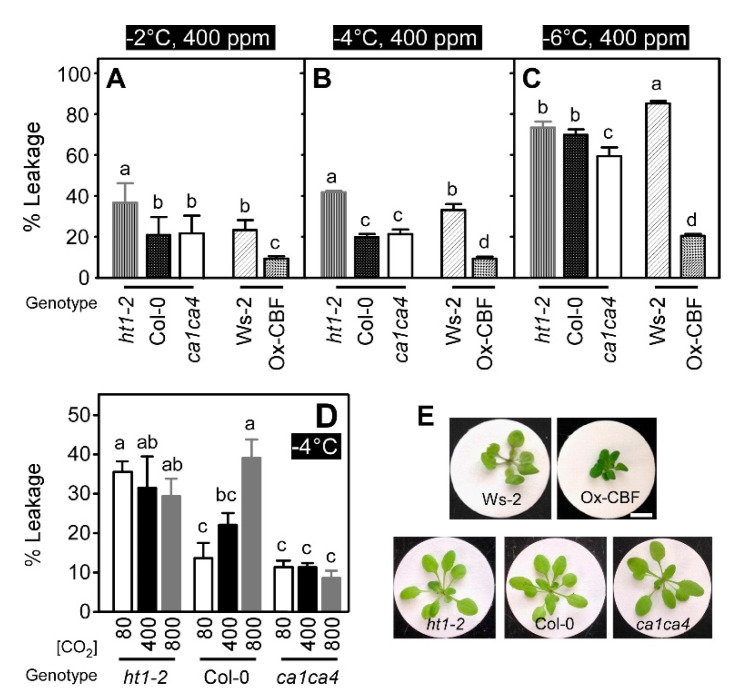
Defects in stomatal responses to CO_2_ alter freezing tolerance in Arabidopsis. Electrolyte leakage was analyzed using Arabidopsis stomatal response mutant (*ht1-2*, *ca1ca4*) ant their wild-type ecotype, Col-0, as well as a CBF3 over-expression plant (Ox-CBF) and its wild-type ecotype, Ws-2. Individual 3.5-week-old plants (**E**) were initially incubated at 4 °C for 3 h at ambient CO_2_. The chamber temperature was then lowered at 1 °C h^−1^ and plants were sampled for electrolyte leakage at −2 (**A**), −4 (**B**), and −6 °C (**C**). Electrolyte leakage under varying CO_2_ levels at −4 °C (**D**) was examined. Electrolyte leakage into deionized water was measured as changes of conductivity and the percentages shown are mean values from three biological replicates and two technical replicates (error bars indicate SEM). Bars labeled with different lowercase letters differed at *p* ≤ 0.05 and differences were determined using One-way (**A**–**C**), Two-way ANOVA (**D**) and Tukey HSD (**A**–**D**).

To confirm that the CO_2_-dependent modulation of CBF-mediated cold signaling in Arabidopsis was positively correlated with freezing tolerance, we examined electrolyte leakage at −2, −4, and −6 °C under ambient CO_2_ condition in the two CO_2_ stomatal response mutants (Figure 4E). Electrolyte leakage from the *ht1-2* mutant was about 2-fold greater (*p* ≤ 0.01) at −2 and −4 °C than that of Col-0 (Figure 4A,B), which is consistent with reduced expression of both *CBF* and downstream CBF target genes in this mutant (see Figure 3A). At −6 °C, however, electrolyte leakage from the Col-0 ecotype and the *ht1-2* mutant were similar (*p* > 0.05) (Figure 4C). Unlike *ht1-2*, electrolyte leakage from the *ca1ca4* double mutant was similar to that of Col-0 at both −2 and −4 °C (*p* > 0.05), but freezing tolerance at −6 °C was enhanced for the double mutant compared to Col-0 (*p* ≤ 0.01) (Figure 4C). Values of EL_50_ (a freezing temperature that produces 50% cell damage) were −5, −4, and −6 °C for the Col-0, *ht1-2* and *ca1ca4* mutants, respectively. This indicates that the *ht1-2* (more closed) and the *ca1ca4* (more open) stomatal mutants were less and more freezing-tolerant than Col-0, respectively. As expected, the Ox-CBF line had the lowest electrolyte leakage at any freezing temperatures (Figure 4A–C).

We further examined CO_2_ modulation of freezing tolerance using the CO_2_ response mutants, *ht1-2* and *ca1ca4* (Figure 4D,E). The percentage of ion leakage at sub-ambient, ambient, and supra-ambient CO_2_ levels was higher in the *ht1-2* mutant compared to the Col-0 at sub-ambient as well as ambient CO_2_ conditions indicating that low CO_2_-dependent freezing tolerance was abolished in the CO_2_ hypersensitive mutant (Figure 4D). The opposite result was observed in the *ca1ca4* mutant in that percentage of ion leakage at sub-ambient, ambient, and supra-ambient CO_2_ levels was lower in the *ca1ca4* mutant compared to the Col-0 at supra-ambient as well as ambient CO_2_ conditions, indicating that CO_2_-dependent freezing tolerance was retained in the CO_2_ insensitive mutant (Figure 4D).

### 2.5. Relationship between CO_2_ and Cold Signaling in Stomatal Movement

**Figure 5:**
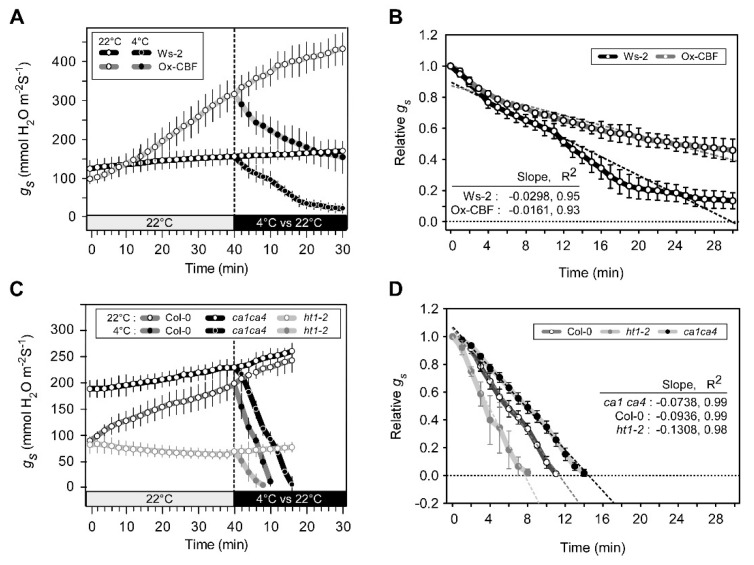
Relationship between CO_2_ and cold signaling in stomatal movement. Time-resolved stomatal conductance (*g_s_*) was measured using 4- to 5-week-old Arabidopsis [Ws-2 and Ox-CBF (**A**,**B**)] and [Col-0, *ht1-2*, *ca1ca4* (**C**,**D**)]. Plants were initially pre-equilibrated at supra-ambient CO_2_ (800 μmol mol^−1^) for 20 min (not shown) followed by 40 min at sub-ambient CO_2_ (80 μmol mol^−1^) at 22 °C (grey box). Subsequently, plants in the sub-ambient CO_2_ treatment were incubated at either 22 or 4 °C (black box) for 30 min. Absolute (**A**,**C**) and relative (**B**,**D**) changes in *g_s_* were measured using five to eight plants (error bars indicate SEM) from two independent experiments.

To further understand the association of CO_2_-induced stomatal movement and the CBF-mediated cold response, we examined time-course changes in stomatal conductance (*g_s_*) using Ox-CBF and its corresponding wild-type plant, Ws-2. CO_2_ stomatal response mutants *ht1-2* and *ca1ca4* were also studied for their stomatal response along with the corresponding wild ecotype Col-0. Plants were initially pre-treated at supra-ambient CO_2_ for 20 min followed by sub-ambient CO_2_ for 40 min. These CO_2_ treatments sequentially induced stomata to close and then open. Plants were then subjected to 4 °C cold treatment while maintaining the sub-ambient CO_2_ treatment. For comparison, stomatal conductance was continuously measured with control plants exposed to 22 °C air temperatures. Whereas the conductance measurements indicated that stomata of Ws-2 wild type were almost completely closed after 30 min of a 4 °C treatment, conductance of Ox-CBF decreased by about 50% after 30 min of exposure to 4 °C (Figure 5A). When stomatal conductance *g_s_* values were normalized, and slopes were calculated for stomatal aperture response to low-temperature treatment, the rates of stomatal closure were about double for Ws-2 compared to Ox-CBF (Figure 5B). Interestingly, when Ox-CBF, a freezing tolerant plant, was kept at sub-ambient CO_2_ at 22 °C, the stomata of Ox-CBF continued to open rapidly over the 30 min of sub-ambient CO_2_ exposure. In contrast, the stomata of Ws-2 opened slowly under this treatment (Figure 5A and Figure S4). The CO_2_ stomatal response mutants *ht1-2* and *ca1ca4* displayed different stomatal responses compared to their corresponding ecotype Col-0. The hypertensive mutant closed its aperture early and insensitive *ca1ca4* closed later than Col-0 at 4 °C. Col-0, *ht1-2*, and *ca1ca4* all closed their stomata after 8-16 min at 4 °C (Figure 5C,D).

## 3. Discussion

The function of stomata is to optimize gas exchange and water relations between the plant and the surrounding atmosphere. Changes in stomatal aperture establish a balance between two vital processes—CO_2_ uptake from the air and water loss from the leaf. Therefore, stomatal movement is an intricately regulated process that involves simultaneously sensing CO_2_ levels in the air, the water potential of the leaf and various other environmental parameters. In the current study, we demonstrated that varying atmospheric CO_2_ altered cold stress signaling in Arabidopsis and that this partly involved the CO_2_ signaling pathway associated with stomatal movement. Supra-ambient CO_2_ decreased the cold-induced expression of three *CBF* genes, and this presumably diminished the expression of downstream cold responsive genes, *KIN1*, *KIN2*, *COR15a*, and *COR5b*. Contrary to the expected results, supra-ambient CO_2_ did not suppress the cold induction of *COR47*, *COR78*, and *ERD10* genes; however, sub-ambient CO_2_ enhanced CBF’s cold-induced expression of all CBF target genes. Therefore, the expression of CBF-dependent downstream cold-responsive genes in Arabidopsis was either fully or partly modulated by CO_2_.

It was previously suggested that freezing injury primarily results from dehydration [38] and that freezing tolerance involves resistance to dehydration [39,40,41]. The partially CO_2_-dependent CBF target genes, *COR47*, *COR78*, and *ERD10*, belong to a gene family that encodes late embryogenesis abundant (LEA) or LEA-related proteins and are involved in mitigating dehydration stress [19,42,43,44,45,46]. LEA proteins enhance freezing tolerance by alleviating damage from dehydration associated with freezing injury [19]. In addition, the fully CO_2_-dependent CBF target genes identified in this study, *KIN1*, *KIN2*, *COR15a,* and *COR15b*, are known to function as antifreeze proteins that enhance the cryostability of the plasma membrane [47,48].

Varying CO_2_ levels during low temperature treatment altered freezing tolerance of the Col-0 ecotype as measured by electrolyte leakage at −2 and −4 °C. This was not significant when electrolyte leakage was measured at −6 °C, at which ion leakage was more than 65%. Electrolyte leakage was enhanced by supra-ambient CO_2_ and the opposite response was observed for low CO_2_ treatments (Figure 2B,C). Therefore, changes in freezing tolerance in the Col-0 ecotype were consistent with the observed changes of *CBF* expression and concomitant changes in the expression of downstream cold responsive genes in response to varying CO_2_ levels described above. Elevated CO_2_ was found to modulate the xylem sap pH, which increases the ABA concentration, affecting several *COR* gene products [2]. However, the precise mechanism by which CO_2_ modulates the low-temperature induction of different groups of CBF-dependent target genes is not clear. The enhanced freezing tolerance observed in this study may be associated with the functions associated with the *CBF* genes studied [1]. The changes in the atmospheric CO_2_ levels did not have a significant effect on the basal expression of the CBF genes measured at room temperature (Figure S3). This suggests that the CO_2_ concentrations in air altered the freezing tolerance of Arabidopsis Col-0 by mediating the expression of *CBF* and various *CBF* target genes.

Low-temperature experiments were performed using CO_2_ stomatal response mutants in the Col-0 genetic background (i.e., the *ht1-2* mutant, hypersensitive to CO_2_ and the *ca1ca4* double mutant, insensitive to CO_2_) to understand the involvement of CO_2_-triggered stomatal movement in the CBF cold signaling pathway in Arabidopsis. Interestingly, *CBF* expression in response to cold treatment was greater in Col-0 than in either stomatal response mutant. In Arabidopsis, the *HT1* gene product has been reported to control stomatal movement in response to CO_2_ [36]. The finding that stomatal closure in the *ht1-2* mutant at ambient CO_2_ conditions resulted in decreased *CBF* expression relative to Col-0 (Figure S2) is consistent with the observed effects of supra-ambient CO_2_ on *CBF* expression in Col-0 (Figure 1A). CO_2_ modulation of cold-induced CBF expression was also evident in the CO_2_ response mutants, *ht1-2* and *ca1ca4*. The increase in cold-induced CBF expression at a sub-ambient CO_2_ level in the wild-type Col-0 (Figure 1A) was abolished in the CO_2_ hypersensitive mutant, *ht1-2* (Figure 3A), while the decrease in cold-induced CBF expression at a supra-ambient CO_2_ level (Figure 1A) was slightly impaired in the CO_2_ insensitive mutant, *ca1ca4*, compared to Col-0 (Figure 3A). Given that sub-ambient CO_2_ triggered stomatal opening and the observed cold-induced expression of *CBF (*Figure 1A), our findings suggest that enhanced stomatal opening, as occurs in the *ca1ca4* double mutant, did not fully implement the CBF-dependent cold signaling pathway in Arabidopsis. Furthermore, the suppression of cold-induced *CBF* expression in both stomatal response mutants implied that βCA1/4 and HT1 function upstream of *CBF* during cold signaling. The HTl protein kinase functions as a major negative regulator in the high CO_2_ stomatal closure pathway. However, these mutants preserve their response to ABA, suggesting that HT1 acts upstream of the convergence of the CO_2_- and ABA-induced pathways [36]. Among CBF-independent cold signaling pathways, the ABA-dependent cold signaling pathway is important and regulates about ~10% of cold responsive genes [15,49].

Furthermore, the expression analysis of seven CBF target genes in the *ht1-2* and *ca1ca4* stomatal response mutants also suggests the involvement of both CBF-dependent and independent responses. Based on the reduced expression of *CBF* genes shown in both the mutants (Figure S2A), it was hypothesized that the cold-dependent expression of all seven CBF target genes would be lower in the *ht1-2* and *ca1ca4* double mutant compared to Col-0. The expression of *KIN1*, *KIN2*, *COR15a* and *COR15b* was reduced, which was consistent with the change in *CBF* expression in the *ht1-2* mutant (Figure S2C). However, the cold-induced expression of *COR47*, *COR78*, and *ERD10* remained unchanged in this mutant relative to Col-0 under ambient CO_2_ (Figure S2B). On the contrary, in *ca1ca4* mutant relative to Col-0, the expression of *COR47, COR78* and *ERD10* in response to low temperature was enhanced, while the expression of *KIN1*, *KIN2*, *COR15a* and *COR15b* in response to low temperature remained similar (Figure S2B,C). One possible explanation is that βCA1/4 may mediate suppression of the cold-induced expression of *COR47, COR78* and *ERD10* in Col-0 and de-repression (or activation) of the expression of *KIN1*, *KIN2*, *COR15a* and *COR15b* via a CBF-independent cold signaling pathway. To explore the possibility of CBF-independent regulation that can be modified by CO_2_, we examined the expression of several CBF-independent cold-responsive genes upon cold treatment in the *ca1ca4* mutant and compared to sub- and ambient CO_2_ treated Col-0 plants. Interestingly, the low temperature increased the expression of *CHS* both in the *ca1ca4* double mutant and in Col-0 in sub-ambient CO_2_, comparable to *CHS* expression changes in the Col-0 plants treated with ambient CO_2_. It was previously reported that a mutation in an Arabidopsis *CHS* gene (*tt4*) decreased anthocyanin biosynthesis [50] and this reduced the capacity of the plant for cold acclimation [51]. Conversely, plants with enhanced levels of anthocyanin and flavonoids had improved cold tolerance [52]. Based on these results, we propose that in Arabidopsis, there exists CO_2_ regulation of both CBF-dependent and -independent cold signaling. In general, CO_2_ modulation of cold induced expression of CBF target genes in the CO_2_ hypersensitive mutant, *ht1-2*, was reduced compared to Col-0 while the opposite was the case in the CO_2_ insensitive mutant, *ca1ca4* (Figure 3B).

Electrolyte leakage analysis in the two stomatal response mutants supports a role for stomatal movement in cold signaling, which may directly or indirectly affect cold-induced gene expression. The *ht1-2* mutant results are consistent with the changes to the CBF-dependent cold signaling data discussed above and showed that electrolyte leakage increased when measured at −2 and −4 °C. Conversely, no differences were observed for electrolyte leakage between the *ca1ca4* double mutant and Col-0 when freezing tolerance was measured at −2 and −4 °C. However, freezing tolerance of the *ca1ca4* mutant exceeded that of Col-0 when measured at −6 °C. The latter finding may be partly due to the enhanced expression of cold responsive genes, such as *COR47*, *COR78*, *ERD10,* and *CHS* in *ca1ca4* relative to Col-0 that was observed in Figure S2B. These observations were reflected in the EL_50_ values, which indicated that the *ca1ca4* mutant was more cold-tolerant and the *ht1-2* mutant was more cold-sensitive than Col-0. These results demonstrate that cold-induced expression of CBFs and their target genes were enhanced, and resulting freezing tolerance was improved when plants were subjected to sub-ambient CO_2_. However, a stomatal response mutant that mimicked low-CO_2_ treatment and possessed opened stomata did not enhance CBF-dependent cold-induced expression relative to Col-0. Additional experiments were performed to further examine CO_2_ modulation of freezing-induced ion leakage using these stomatal response mutants, which demonstrated that increase in freezing tolerance at sub-ambient CO_2_ condition in the Col-0 was abolished in the CO_2_ hypersensitive mutant, *ht1-2*, while the opposite, decrease in freezing tolerance at supra-ambient CO_2_ condition, was impaired in the CO_2_ insensitive mutant, *ca1ca4* (Figure 4D).

To further understand the relationship between CO_2_-induced stomatal movement and CBF-mediated cold response, we measured *g_s_* on the Ox-CBF line and two CO_2_ stomatal response mutants along with their corresponding wild-type plants (Figure 5A–D and Figure S4). The conductance data demonstrated that low temperature (4 °C) induced stomatal closure even though Arabidopsis plants were treated with sub-ambient CO_2_. This finding demonstrates that rapid cold signaling in response to low temperature was capable of overriding the CO_2_ signaling involved in stomatal movement. Low temperature diminishes plant survivability; therefore, it is likely that severe stress would induce stomatal closure even when sub-ambient CO_2_ levels signal stomatal opening. However, it is important to note that measurements of *g_s_* using Ox-CBF, i.e., the freezing tolerant line, showed that stomata were about half opened 30 min after 4 °C cold treatment was initiated. Furthermore, low temperature-induced stomatal closure rates were delayed in a cold tolerant stomatal response mutant, *ca1ca4,* and accelerated in a cold sensitive mutant, *ht1-2*, compared to Col-0 (Figure 5C and D). Stomatal conductance of the Ox-CBF (Overexpressed CBF3) mutant demonstrated a significant response in stomatal opening to sub-ambient CO_2_ levels at 22 °C when compared to other plants, reflecting CO_2′_s effect on enhanced freezing tolerance observed in the mutant at low temperatures (−6 °C) (Figure 2D).

## 4. Conclusions

**Figure 6:**
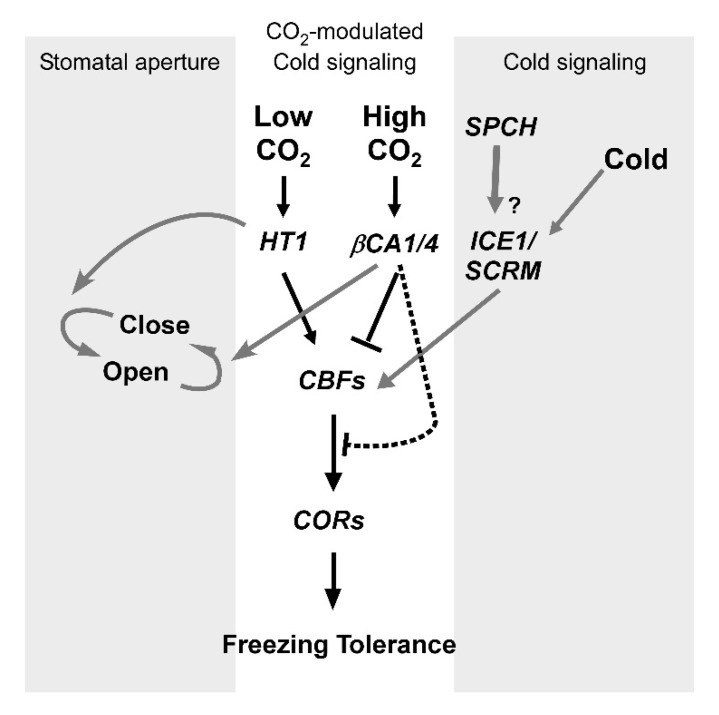
A schematic diagram for CO_2_-mediated CBF-dependent cold signaling and freezing tolerance in Arabidopsis. Black arrows indicate a proposed CO_2_ modulated cold signaling pathway (**center**). Grey arrows represent signaling pathways involved in stomatal movement (**left**) and freezing tolerance (**right**). This model proposes that CO_2_-modulated CBF-dependent cold signaling is mediated by βCA1/4 and HT1. In response to supra-ambient CO_2_, βCA1/4 suppresses HT1, which decreases both CBF-mediated cold signaling and freezing tolerance, while suppression of HT1 by βCA1/4 is reduced in response to sub-ambient CO_2_, and this enhances CBF-mediated freezing tolerance. In addition, βCA1/4 mediate suppression of CBF-independent expression of downstream cold-responsive genes. A potential new connection based on our preliminary results is shown as a dotted line. The question mark indicates that the gene responsible for the indicated pathway has not yet been definitely identified.

In summary, this study demonstrated that the induced gene expression due to varying CO_2_ and low temperatures had a more significant impact on cold tolerance (electrolyte leakage) than the actual stomatal aperture. A general model for interactions of regulatory pathways for stomatal movement, CO_2_, and cold-induced gene expression is summarized in Figure 6. It is proposed that two β-carbonic anhydrases, i.e., βCA1/4, and a protein kinase, *HT1*, mediate CO_2′_s effects on low temperature-induced *CBF* expression in Arabidopsis. This alters the expression of downstream cold-responsive genes. The model suggests that in response to supra-ambient CO_2_, βCA1/4 suppresses HT1, which decreases both CBF-mediated cold signaling and freezing tolerance. Conversely, in response to sub-ambient CO_2_, suppression of HT1 by βCA1/4 is reduced, enhancing CBF-mediated freezing tolerance.

We also propose the existence of βCA1/4-dependent repression and de-repression of downstream cold-responsive genes that likely occur in a CBF-independent manner. Interactions among genes upstream of *CBF* (e.g., *ICE1/SCRM* and *SPCH*) and the CO_2_ signaling pathway in Arabidopsis are currently unknown. However, it is interesting to note that ABA-dependent cold signaling also occurs in plants and that ABA levels increase in plants exposed to low temperatures [15]. ABA is also an essential factor in stomatal closure [49]. Unraveling the role of ABA signaling in cold response may provide additional links between CO_2_ and cold signaling pathways. Our findings demonstrated in the current study will add another layer of the complex regulation of the plant’s response to environmental stimuli. Studying stomatal responses to CO_2_ under cold stress will also help us to understand plant cold-responsive mechanisms under climate change conditions. This information can be translated into essential crops to enhance freezing tolerance and productivity.

## 5. Materials and Methods

### 5.1. Plant Materials and Growth Conditions

*Arabidopsis thaliana* L. Heynh wild type and mutant plants were grown in controlled environment chambers (model M-15, EGC corp., Chagrin Falls, OH, USA) under ambient 400 ppm CO_2_ and day/night temperatures of 22 °C/18 °C. Two wild type (Col-0 and Ws-2) and two mutant lines (*ca1ca4* and *ht1-2*) as well as one overexpression line (Ox-CBF) were used in this study. The description of Arabidopsis plants used in the study are listed in Table S1. The photoperiod was 16 h light and 8 h dark with a photosynthetic photon flux density of 110 ± 10 µmol m^−2^ s^−1^. Relative humidity was not controlled but was 70 to 80% during the light period_._ Individual 10 day-old seedlings were transplanted to 1.5 × 1.5 inch pots filled with 85% peat mixture (MetroMix, Morgantown, PA, USA) and vermiculite (Grace Construction Products, Cambridge, MA, USA). Pots were irrigated from the bottom with water and received one quarter strength nutrient solution once or twice weekly.

### 5.2. Low Temperature and CO_2_ Treatments

Four to twelve pots containing a single, 3.5-week old plant were placed in a 45 × 37 × 12 cm deep incubation chamber that was clamped shut with a clear acrylic plastic lid. An adhesive foam seal (Armaflex Insulation Tape (9.1 × 3 × 50 mm), Armacell LLC., Mebane, NC, USA) was placed between the top of the incubation chamber and the lid to minimize air exchange. Illumination (115 ± 10 µmol m^−2^ s^−1^ PPFD) was provided by a bank of fluorescent lamps (model 48T10/CW, 110W, VHO, General Electric, Buffalo, NY, USA) located 1 m above the upper surface of the incubation chamber. Control plants were enclosed in the sealed incubation chamber at 22 °C and kept in the same controlled environment chamber used for plant growth. Modified air of known CO_2_ concentrations and temperature was pumped through the entrance and exit holes in the sealed lid of the incubation chamber at a flow rate of 3 to 4 l min^−1^. The CO_2_ concentration of the air entering and exiting the incubation chamber was monitored with an infrared gas analyzer (model WMA-3, PP systems, Haverhill, MA, USA). Supra-ambient CO_2_ was obtained by slowly injecting 1% CO_2_ into the airstream entering the incubation chamber. Standard gases were from Airgas (Hyattsville, MD, USA), and the other contents of the cylinder were approximately 79% N_2_ and 20% O_2._ Sub-ambient CO_2_ was obtained by scrubbing the air entering the incubation chamber with soda-lime and then injecting known amounts of CO_2_ from a standard gas cylinder via a micro metering valve. The three CO_2_ treatments were 80, 400, and 800 ± 10 ppm (µmole CO_2_ mole^−1^ air). Low temperature treatments were initiated by placing the incubation chamber, gas cylinder, CO_2_ scrubber and infrared gas analyzer in a walk-in cold room maintained at 4 °C. After an equilibration period to attain desired CO_2_ levels and treatment temperatures, plants were placed in the incubation chamber and the lid was rapidly sealed. The time to attain the desired CO_2_ concentration within the incubation chamber after sealing the plants inside was about 5 to 10 min for a small chamber (for transcript analysis) and 15 to 20 min for a big incubation chamber (for freezing tolerance assay). Illumination was as described above and temperatures within the incubation chamber were measured with a fine wire thermocouple. Temperature and CO_2_ treatments were maintained for 2h before the incubation chamber was quickly opened and rosette leaves were collected into liquid N_2_ for transcript analysis and stored at −80 °C until analysis.

### 5.3. Transcript Analysis

Frozen rosette leaves were ground to a fine powder under liquid N_2_ using a sterile mortar and pestle. Total RNA was extracted from 30 mg fresh weight of frozen tissue and cDNA synthesis was performed as previously described [53,54]. Up to 5 µl of diluted cDNA was used in qPCR reactions contained in a total volume of 20 µl. The gene-specific primers for reference genes *Actin 2* (*ACT2*) and *Transcription Elongation Factor* (*TEF*) and for *CBF1-3*, *COR78*, *COR15a*, and *ERD10* from Arabidopsis were previously described [55]. All other primer pairs were designed using the Primer 3 program and primer sequences used for qPCR are listed in Table S2. qPCR was performed as previously described [54] and relative transcript abundance was measured by normalizing the expression level of each gene of interest using the expression of reference gene *ACT2*. *ACT2* and *TEF* showed the same expression pattern, but % PCR efficiency of *ACT2* was greater than *TEF* (97 vs. 94% respectively); therefore, *ACT2* data were used for normalization. The presence of a single PCR product was further verified by dissociation analysis. Three independent experiments were performed with three to four plants per treatment. Significant differences were determined using a one-way or two-way ANOVA procedure in combination with Tukey HSD (StatView 5.0, Mountain View, CA, USA).

### 5.4. Electrolyte Leakage Freeze Test

Electrolyte leakage tests were performed on excised leaves as previously described [56,57] and freezing tolerance was determined by the method of Sukumara and Weiser [58]. Four or more excised Arabidopsis leaves from 4-week old plants were placed in 13 × 100 mm glass culture tubes (Corning Inc., Corning, NY, USA), which were maintained at 4 °C for 3 h in a low-temperature chamber (LHU-113 Temperature & Humidity Cabinet, ESPEC Corp., Osaka, Japan). Freezing treatments were initiated by adding a few ice chips to each tube. Following a 3 h equilibration period, the chamber temperature was lowered manually by 1 °C h^−1^. Samples were withdrawn from the temperature chamber after plants were exposed to −2, −4 and −6 °C for 1h in descending order. The harvested samples were immediately stored on ice until the end of the experiment and were then thawed overnight in a cold room at 4 °C. To estimate electrolyte leakage, 2 mL of distilled water was added to each tube, and the samples were gently shaken in a water bath (Model G76D, New Brunswick Scientific Co., Inc., Edison, NJ, USA) at room temperature for 3 h. The resulting solution’s electrical conductivity was measured using a conductivity meter (Model 35, YSI, Yellow Springs, OH, USA). A value for 100% leakage was obtained by freezing the tested samples at −80 °C for 1 h before extraction. Levels of CO_2_ within the temperature chamber were controlled during the freezing treatment, as described above, using access ports located on the chamber’s side. The air within the chamber also was circulated with an internal fan to ensure proper mixing. Significant differences were determined using a one-way or two-way ANOVA procedure in combination with Tukey HSD (StatView 5.0, Mountain View, CA, USA).

### 5.5. Stomatal Conductance Determinations

Stomatal conductance (*gs*) was measured using a portable Photosynthesis System (model 6400, Open System 4.0, Li-Cor Inc., Lincoln, NE, USA) using a 0.85-dm^3^ natural sun-lit cuvette. Experiments were performed with single, attached leaves of wild type Arabidopsis, i.e., ecotypes Col-0, Ws-2, and related mutants. All measurements were performed in growth chambers matching those used for plant growth, and irradiance was provided by fluorescent bulbs as described above. Treatments were initiated 3 h after the start of the light period with plants initially maintained at 22 °C, 110 μmol m^−2^ s^−1^ PPFD, and at 400 ppm CO_2_. Plants were first exposed to 800 ppm CO_2_ for 20 min before being switched to low CO_2_ (80 ppm) for 40 min. Half of the plants were then transferred to 4 °C for 30 min, and the remaining plants served as ambient temperature controls. Data were collected continuously from pre-treatment to the end of the experiment. Conductance values (*gs*) were calculated by the Photosynthesis System and were collected using five to eight plants from two independent experiments.

## Supplementary Materials

Supplementary Materials can be found at https://www.mdpi.com/1422-0067/21/20/7616/s1. Figure S1. Cold-induced expression of *CHS, ATHB2,* and *RD22* genes in *ca1ca4* mutant and Col-0 plants was determined under sub-ambient CO_2_ (80 μmol mol^−1^) or ambient CO_2_ (400 μmol mol^−1^) conditions. Figure S2. The low temperature expression of CBFs and downstream CBF-dependent genes in ambient CO_2_ was determined in two Arabidopsis CO_2_ stomatal response mutants. Figure S3. Basal expression of *CBF* genes at room temperature under sub-ambient (80 μmol mol^−1^), ambient (400 μmol mol^−1^), and supra-ambient CO_2_ (800 μmol mol^−1^) atmosphere conditions was examined. Figure S4. Stomatal opening in response to low CO_2_ treatments in Ox-CBF was compared to that in Ws-2. Table S1. Information on Arabidopsis ecotypes and mutants used in the study. Table S2. The list of primers used in this study.

## Abbreviations

*ht1-2*	*high leaf temperature 1-2*
*ca1ca4*	*β-carbonic anhydrase 1 and 4*
CRT/DRE	C-repeat/dehydration response element
*COR*	*Cold-responsive*
*ERD*	*Early dehydration inducible*
*RD*	*Responsive to dehydration*
*KIN*	*Cold-inducible*
*ICE1*	*Inducer of CBF Expression 1*
*SCRM*	*SCREAM*
*SPCH*	*SPEECHLESS*
*ACT2*	*Actin 2*
*g_s_*	Stomatal conductance
*ATHB2*	*Arabidopsis thaliana homeobox 2*
*CHS*	*Chalcone synthase*
*LEA*	*late embryogenesis abundant*
