# Supplementary material for: Varying Atmospheric CO2 Mediates the Cold-Induced CBF-Dependent Signaling Pathway and Freezing Tolerance in Arabidopsis
Source: Int J Mol Sci. 2020 Oct 15;21(20):7616. doi: 10.3390/ijms21207616 (PMC7593905; doi:10.3390/ijms21207616)
Supplement: Supplementary file 1 [file ijms-21-07616-s001.docx]

Supplemental file

**Varying atmospheric CO_2_ mediates the cold-induced CBF-dependent signaling pathway and freezing tolerance in Arabidopsis**

Jinyoung Y. Barnaby^1,2,^*, Joonyup Kim^3^, Mura Jyostna Devi^1^, David H. Fleisher^1^, Mark L. Tucker^3^, Vangimalla R. Reddy^1^ and Richard C. Sicher^1^

^1^ Adaptive Cropping Systems Laboratory, Agricultural Research Service, USDA, Building 001, 10300 Baltimore Ave., Beltsville, MD 20705 USA; [jyostna.mura@usda.gov](mailto:jyostna.mura@usda.gov) (M.J.D.); [david.fleisher@usda.gov](mailto:david.fleisher@usda.gov) (D.H.F.); [Vangimalla.reddy@usda.gov](mailto:Vangimalla.reddy@usda.gov) (V.R.R.); [rsicher1981@gmail.com](mailto:rsicher1981@gmail.com) (R.C.S.)

^2^ Dale Bumpers National Rice Research Center, Agricultural Research Service, USDA, Building 001, 10300 Baltimore Ave., Beltsville, MD 20705, USA; Current address of [Jinyoung.barnaby@usda.gov](mailto:Jinyoung.barnaby@usda.gov) (J.Y.B.)

^3^ Soybean Genomics and Improvement Laboratory, Agricultural Research Service, USDA, Building 006, 10300 Baltimore Ave., Beltsville, MD 20705, USA; [joonyup.kim@gmail.com](mailto:joonyup.kim@gmail.com) (J.Y.K.); [mark.tucker@usda.gov](mailto:mark.tucker@usda.gov) (M.L.T.)

***** Correspondence: [Jinyoung.barnaby@usda.gov](mailto:Jinyoung.barnaby@usda.gov); Tel.:+1-301-504-8436

Received: 01 September 2020; Accepted: 13 October 2020; Published: date


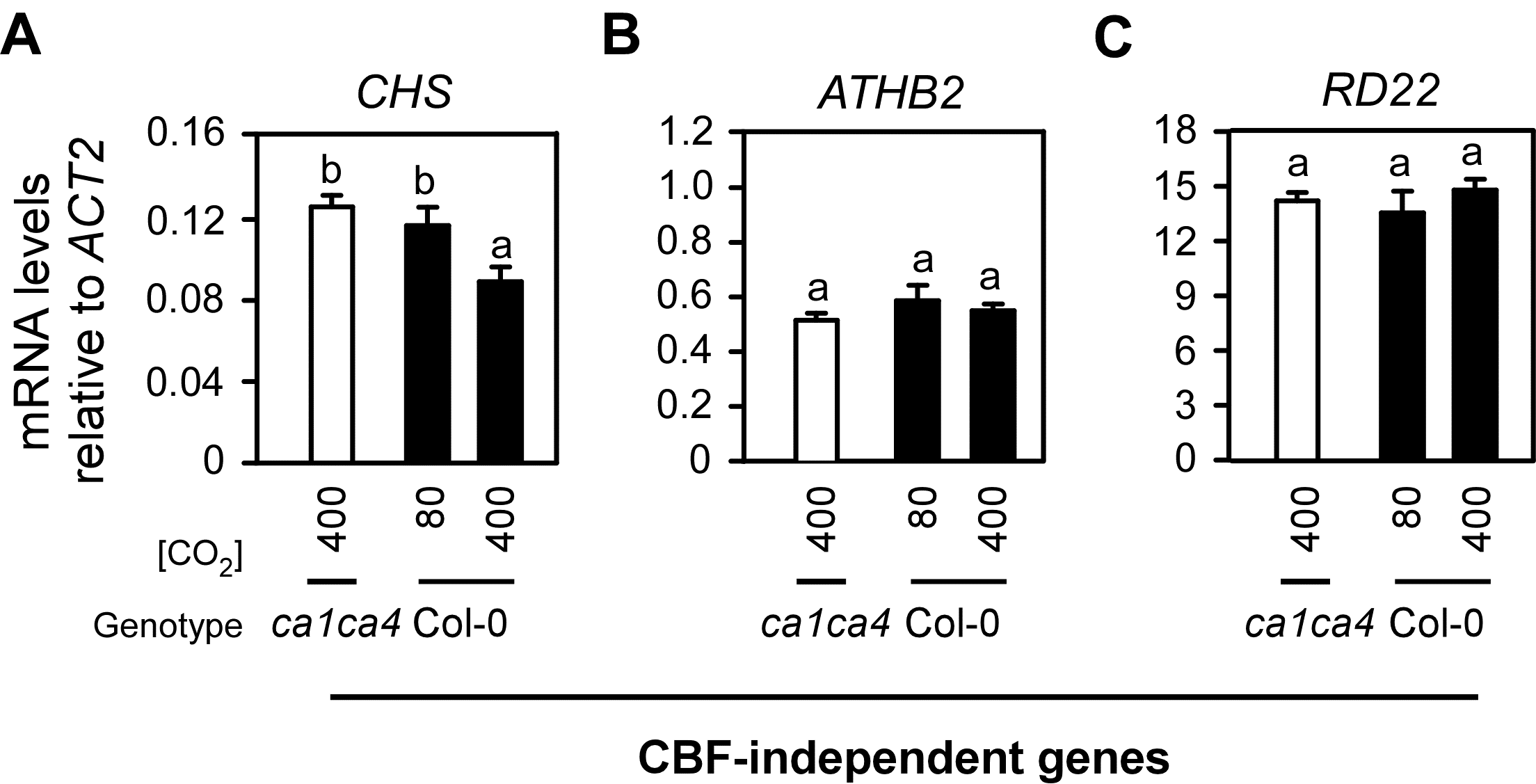


**Supplemental Figure S1.** **Cold-induced expression of *CHS* gene was positively correlated with that of partially CO_2_-dependent CBF target genes.** Transcript abundance was determined after 2 h of induction at 4 °C using 3.5 week-old *ca1ca4* mutant and Col-0 plants under either sub-ambient CO_2_-treated (80 μmol mol^-1^) or ambient CO_2_-treated (400 μmol mol^-1^). The transcript levels of *CHS* (**A**), *ATHB2* (**B**), and *RD22* (**C**) were measured by q-PCR. Measurements of transcript abundance and other experimental details were as described in Figure 1.

**
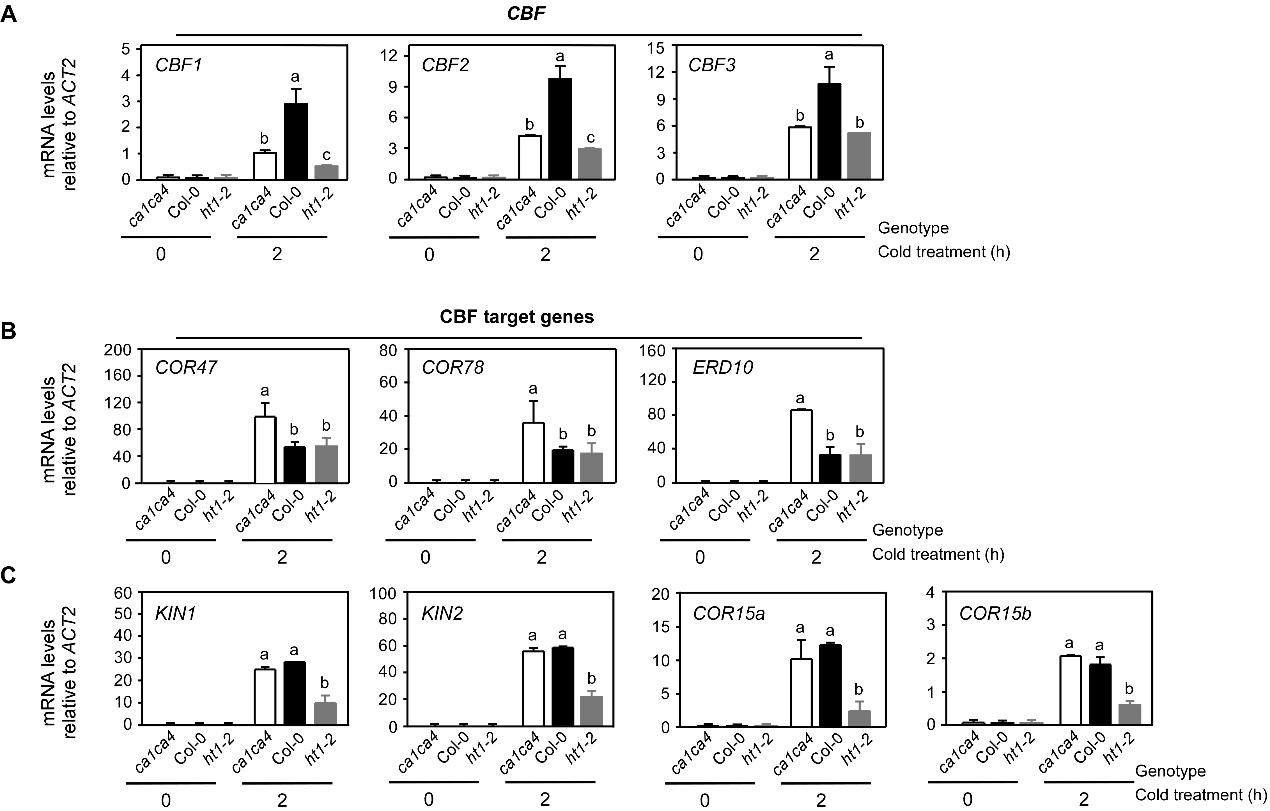
**

**Supplemental Figure S2.** **The low temperature expression of CBFs and downstream CBF-dependent genes in ambient CO_2_ was altered in two Arabidopsis CO_2_ stomatal response mutants.** Transcript abundance was determined using Arabidopsis (Col-0) plants and two stomatal response mutants that were hypersensitive or insensitive to CO_2_ (i.e. *ht1-2* and *ca1ca4*, respectively). Individual 3.5-week-old plants were exposed to 4 °C temperature for 2 h under ambient CO_2_ conditions. Measurements of transcript abundance and other experimental details were as described in Fig. 1.

**
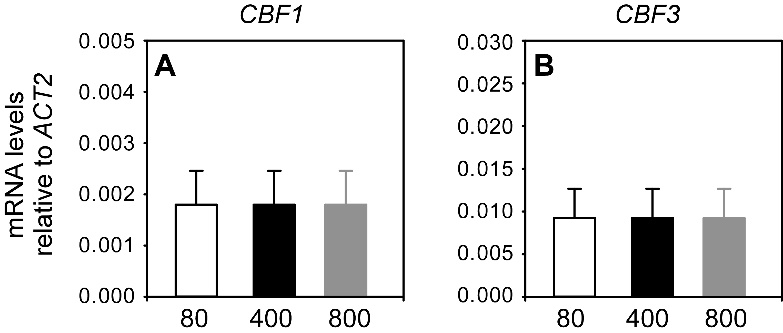
**

**Supplemental Figure S3. CO_2_ does not affect the basal expression level of CBFs under normal conditions (no cold treatment).** Individual 3.5 week-old Arabidopsis plants (Col-0) were exposed to room temperature (22 °C) for 2 h with sub-ambient, ambient, or supra-ambient CO_2_, (80, 400 and 800 μmol mol^-1^, respectively). Transcript abundance of two *CBF* genes (*CBF1* and *CBF3*) was determined by q-PCR. The expression of measured transcripts was normalized to *ACT2*. Values are means of three biological replicates and two technical replicates (error bars indicate SEM).


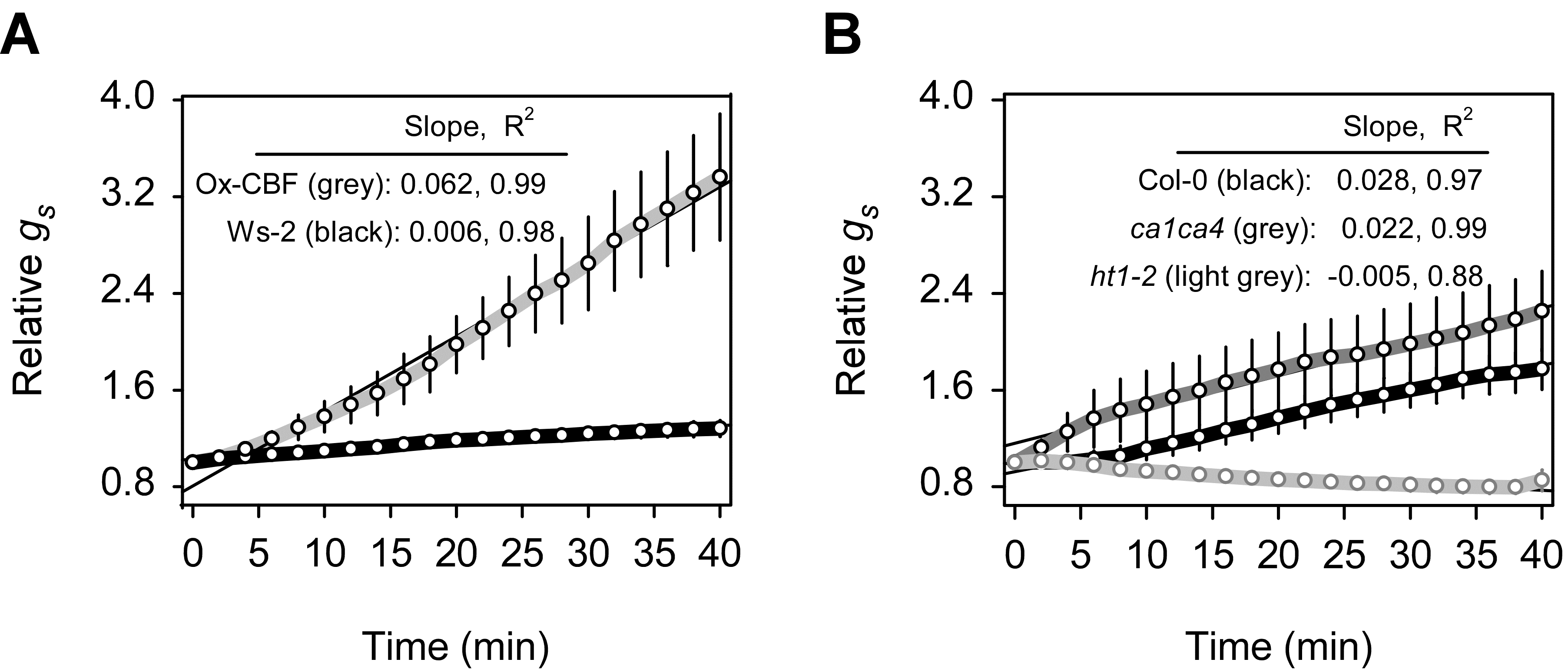


**Supplemental Figure S4.** **Stomatal opening in response to low CO_2_ treatments was greatly accelerated in Ox-CBF compared to that in Ws-2.** Time-resolved stomatal conductance (*g_s_*) was measured using 4- to 5-week-old Arabidopsis [Ws-2 and Ox-CBF (**A**)] and [Col-0, *ht1-2*, *ca1ca4* (**B**)]. Plants were initially pre-equilibrated at 22°C for 20 min with supra-ambient CO_2_ (800 μmol mol^-1^) (not shown) followed by 40 min at sub-ambient CO_2_ (80 μmol mol^-1^). Relative changes of *g_s_* were measured using five to eight plants (error bars indicate SEM) from two independent experiments.

**Table S1.** **Information on Arabidopsis ecotypes and mutants used in the study**

| **Name** | **Accession type** | **Altered gene(s)** | **Function** |
| --- | --- | --- | --- |
| *Col-0* | Ecotype | - | - |
| *Ws-2* | Ecotype | - | - |
| *CalCa4* | Mutant (Col-0) | β-Carbonic anhydrase 1 and 4 | Insensitive to CO_2_ |
| *htl-2* | Mutant (Col-0) | High leaf temperature 1-2 | Hypersensitive to CO_2_ |
| *Ox-CBF* | Mutant (Ws-2) | Over-expresses *CBF3* | Freezing tolerant |

**Table S2. The list of primers used in this study**

| **Name** | **Sequence (5'-3')** | **Tm (°C)** |
| --- | --- | --- |
| ACT2-F | TCAGATGCCCAGAAGTGTGTT | 56.5 |
| ACT2-R | CCGTACAGATCCTTCCTGATAT | 53.3 |
| CBF1-F | GGCCGTAAGAAGTTTCGTGA | 55.1 |
| CBF1-R | ATCGTCTCCTCCATGTCCAG | 56.1 |
| CBF2-F | AACTCCGGTAAGTGGGTGTG | 57 |
| CBF2-R | CGGCGTATAAATAGCCTCCA | 54.3 |
| CBF3-F | ACAGAGGAGTTCGTCGGAGA | 57.3 |
| CBF3-R | ACCAACGTCTCCTCCATGTC | 56.8 |
| COR47-F | AGCGATGAAGAAGGTGAGGA | 55.7 |
| COR47-R | ACACTGGTACCGGGATGGTA | 57.6 |
| COR78-F | CCAGCAGCACCCAGAAGAA | 57.7 |
| COR78-R | TCATGCTCATTGCTTTGTCCAT | 55.3 |
| ERD10-F | AGCCAAGTCTCCTCGACAAA | 56.2 |
| ERD10-R | TACATCATCACCCCCTGGTT | 55.7 |
| COR15a-F | GGCCACAAAGAAAGCTTCAG | 54.5 |
| COR15a-R | CTTGTTTGCGGCTTCTTTTC | 53 |
| COR15b-F | CACAACGTAGGAGCAAGCA | 55.4 |
| COR15b-R | GAGGATGTTGCCGTCACTTT | 55.5 |
| KIN1-F | TGTCAGAGACCAACAAGAATGC | 55.3 |
| KIN1-R | CCGCATCCGATACACTCTTT | 54.8 |
| KIN2-F | ACCAACAAGAATGCCTTCCA | 54.8 |
| KIN2-R | ACTGCCGCATCCGATATACT | 55.9 |
| CHS-F | CGTGTTGAGCGAGTATGGAAAC | 56.1 |
| CHS-R | TGACTTCCTCCTCATCTCGTCTAGT | 58.2 |
| ATHB2-F | GAGGTAGACTGCGAGTTCTTACG | 56.7 |
| ATHB2-R | GCATGTAGAACTGAGGAGAGAGC | 56.9 |
| RD22-F | CCGGTAAAAGAACCGACGTA | 54.5 |
| RD22-R | AAAGGGTTTGCTCCTGGTTT | 55 |

| 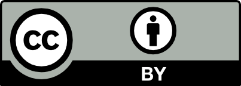 | © 2019 by the authors. Submitted for possible open access publication under the terms and conditions of the Creative Commons Attribution (CC BY) license (http://creativecommons.org/licenses/by/4.0/). |
| --- | --- |
